# A Case of Peutz-Jeghers Syndrome Complicated With Hirschsprung Disease

**DOI:** 10.7759/cureus.95100

**Published:** 2025-10-21

**Authors:** Saori Murakawa, Masato Ogawa, Aoi Taku, Genshiro Esumi, Masanori Hisaoka, Takayuki Hoshina

**Affiliations:** 1 Department of Pediatrics, University of Occupational and Environmental Health, Fukuoka, JPN; 2 Department of Pediatrics, Kyushu Rosai Hospital, Fukuoka, JPN; 3 Department of Pediatric Surgery, University of Occupational and Environmental Health, Fukuoka, JPN; 4 Department of Pathology and Oncology, University of Occupational and Environmental Health, Kitakyushu, JPN

**Keywords:** chronic constipation, gastrointestinal disease, genetic disorder, hirschsprung disease, peutz-jeghers syndrome

## Abstract

We report the first documented case of Peutz-Jeghers syndrome (PJS) complicated by Hirschsprung disease (HD). Despite the patient having intractable constipation since the neonatal period, the diagnosis of HD was significantly delayed due to the earlier diagnosis of PJS in infancy, which masked the presence of HD. This case highlights the importance of considering the possibility of an additional hereditary disease when a patient with a known hereditary gastrointestinal disease develops gastrointestinal symptoms that are atypical for that disease.

## Introduction

Peutz-Jeghers syndrome (PJS) is a rare genetic disorder that causes hamartomatous polyposis throughout the gastrointestinal tract, excluding the esophagus, and distinctive cutaneous mucosal pigmentation of the eyelids and oral mucosa. Enlarged polyps cause small bowel obstruction-related abdominal pain, intussusception of the small bowel, and gastrointestinal bleeding [1]. PJS also carries a high risk of cancer development in the gastrointestinal tract and other organs [1].

Hirschsprung disease (HD), characterized by a lack of ganglia in the myenteric and submucosal plexuses of the gut, is a representative cause of intestinal obstruction in neonates and infants. The symptoms of HD vary with the severity of the condition and are sometimes not apparent until later in life [2].

HD is also considered a genetic disorder, although it may not be caused by monogenic mutations. It is not rare to be complicated by two genetic disorders; however, patients with both PJS and HD have not been reported.

We herein report a patient with PJS complicated by HD. It took a long time to recognize that the intractable constipation the patient had was caused by HD, because the patient had already been diagnosed with PJS, a gastrointestinal tract disorder, during infancy.

## Case presentation

**Figure 1:**
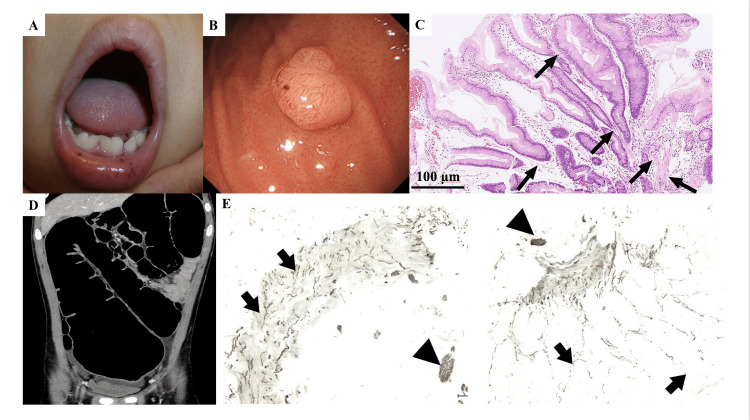
Clinical findings, imaging and histopathological findings in the patient. (A) Mucosal pigmentation on the lip, (B) Gastrointestinal endoscopy showed a polyp in the stomach, (C) Histopathological finding of the gastric polyp showing a hyperplastic foveolar epithelium focally supported by smooth muscle bundles (arrow) in an edematous stroma (hematoxylin and eosin staining, original magnification ×10), (D) Distention of the entire colon without obvious obstruction was indicated on abdominal computed tomography, (E) The finding of acetylcholinesterase staining in rectal mucosa (original magnification ×100). An increase in acetylcholinesterase-positive nerve fibers in the mucosal muscularis mucosae and the mucosa intrinsic layer (black arrow) as well as the thick bundle of nerve fibers in the submucosal layer (triangle) was observed. Ganglion cells were absent.

A 13-year-old boy visited our hospital because of abdominal distension, pain, and vomiting. He had been clinically diagnosed with PJS due to mucosal pigmentation on his lip at six months old (Figure 1A) and multiple polyps in the stomach, small intestine, and rectum at three years old (Figure 1B). The polyps showed hamartomatous hyperplasia of the mucosal epithelium, indicating typical PJS polyps (Figure 1C). A genetic analysis was not performed because of the lack of consent from his parents. None of his family members had any gastrointestinal disorders. The patient had been treated with magnesium oxide for intractable constipation since the neonatal period and occasionally complained of abdominal pain that required hospitalization but resolved without specific treatment. These symptoms were considered to be caused by the concomitant obstruction of the gastrointestinal tract due to PJS polyps. A physical examination at the time of the hospital visit showed abdominal distention and tenderness. A rectal examination revealed no anal stenosis. Abdominal computed tomography indicated marked dilatation of the colon and gas retention, without obvious obstruction (Figure 1D). Lower gastrointestinal endoscopy showed no progression of enlarged polyps. The symptoms spontaneously improved without any specific treatments. HD was suspected because of strong contraction of the anal sphincter and persistent and intractable constipation. The patient underwent a biopsy of the rectal mucosa in a higher-level medical institution. The biopsied specimens showed an increase in acetylcholinesterase-positive nerve fibers in the mucosal muscularis mucosae and the mucosal intrinsic layer, as well as the absence of ganglion cells (Figure 1E). The patient was diagnosed with PJS complicated by HD and underwent radical HD surgery. He continues to take oral medication for constipation.

## Discussion

PJS is a disease with an extremely rare incidence rate of one in 8,300 to 200,000 people. The prevalence of this disease is estimated to be approximately one in 50,000 to 200,000 people [3]. As previously mentioned, PJS is characterized by distinctive skin findings, gastrointestinal symptoms due to polyposis, and a future risk of cancer development. PJS is caused by mutations in the* STK11* gene located on chromosome 19. This gene encodes a serine/threonine kinase that plays a crucial role in regulating cell growth, polarity, and metabolism. The PJS mutation is typically inherited in an autosomal dominant pattern. This mutation causes loss of function in the *STK11 *gene, leading to the aforementioned pathologies [4]. 

HD is also a rare and complex congenital disorder with an incidence rate of one in 5,000. It is characterized by the absence of enteric neurons in the digestive tract, causing functional colonic obstruction. To date, more than 20 genes, including ret proto-oncogene (*RET*), endothelin B receptor gene (*EDNRB*), and paired-like homeobox 2B gene (*PHOX2*), have been reported to be involved in the development of HD [5]. In addition, syndromes associated with HD include trisomy 21, Mowat-Wilson syndrome, congenital central hypoventilation syndrome, Shah-Waardenburg syndrome and cartilage-hair hypoplasia [5].

However, there are no reports of PJS or* STK11*, its responsible gene, being associated with the development of HD. A mutation in the *STK11* gene is the only known cause of PJS [6]. However, some patients with PJS do not harbor *STK11* mutations [6]. Our patient may have had a mutation other than one in the *STK11* gene, although a genetic analysis was not performed. Alternatively, the *STK11 *gene may be involved in the development of HD, or HD and PJS may have occurred coincidentally in this patient. HD typically manifests in the neonatal period or infancy with symptoms such as constipation and delayed passage of meconium. In contrast, HD patients with aganglionic segments limited to the rectum or rectosigmoid are occasionally diagnosed after infancy [2]. Thus, HD should be considered for patients with chronic constipation from birth. Our patient had chronic constipation that required treatment with magnesium oxide. However, we speculated that the intractable constipation was caused by an obstruction of the gastrointestinal tract due to polyps, because PJS had been diagnosed prior to HD. A previous study reported that the median age at the onset of gastrointestinal symptoms in PJS was approximately 13 years old [7]. This result is unusual for our patient with PJS, because he has had intractable constipation since the neonatal period. In addition, constipation is a rare gastrointestinal symptom in PJS patients [1].

## Conclusions

We report a case of concomitant gastrointestinal diseases, HD and PJS. While some HD cases show no genetic abnormalities, most do exhibit them, and PJS also involves* STK11 *gene abnormalities. That is, both HD and PJS can be said to be associated with genetic abnormalities. Although genetic analysis was not performed in this case, it was suggested that PJS and its responsible gene,* STK11*, may have been associated with the development of HD, that our patient may have had mutations other than the *STK11* gene, and that HD and PJS may have developed in this patient by chance. It is necessary to consider the possibility of another hereditary disease when a patient with a hereditary gastrointestinal disease develops gastrointestinal symptoms that are atypical for that disease.
